# Academic-faculty environment and graduate employability: variation of work-readiness perceptions

**DOI:** 10.1016/j.heliyon.2022.e09117

**Published:** 2022-03-16

**Authors:** Bianca Ifeoma Chigbu, Fhulu. H. Nekhwevha

**Affiliations:** Department of Sociology, University of Fort Hare, P.B. X1314, Alice, 5700, South Africa

**Keywords:** Prospective graduates' employability, University environment, Faculty influence, Higher education institutions, Skills, Work readiness

## Abstract

Graduates with a high level of competence can cope better with the disequilibria triggered by events such as shifting labor processes and job transitions. This study examined the relationship between prospective graduates' perceived job preparedness and the university's role in preparing students for the workforce. A sample of 335 South African university students was used. We discovered that skill inequalities vary greatly between faculties. Collegiate skill preparation of students for the workplace can explain the disparities in graduate skill development and recruitment opportunities. Universities and their faculties must reimagine themselves as the primary drivers of graduate skill development and expand pipelines for the most vulnerable prospective graduates to contribute to global skills need.

## Highlights

• The levels of skill disparity range significantly between faculties.
• An exclusionary approach to university skills training for the job market may explain the disparity in skill development among university faculties.
• Learning is the result of a complex interplay between educational institutions and the performance of students.
• The university faculty must address existing educational issues and become more proactive in the changing labor market.

## 1 Introduction

Organizations are heavily reliant on providers of education to deliver employment-ready graduates. A workforce that is skilled attracts investors to a country like South Africa. This attraction, in turn, results in the growth of the economy, thereby eliminating a key obstacle for investment and development in South Africa. The providers of education must be urgently enhanced since higher education graduates’ ability to work in a global, diverse background seems to be ill-prepared (van Nieuwenhuizen, 2012; Kapareliotis et al., 2019; Ohei et al., 2019; Prikshat et al., 2019; Okolie et al., 2020).

The issue worldwide is that investing in tertiary education must expand the source of human capital stock's national economic well-being, which contributes significantly to the economy. Higher institutions are expected to nurture the desired educational output and employability needed in the economy (Knight and Yorke, 2003; Shivoro et al., 2018; Ndebele and Ndlovu, 2019). According to Robertson (2011), an economy is a group of individuals who employ understanding and capability cooperatively to produce an economic privilege far superior to that which existed formerly.

Lack of employability skills is noticeable in a country like South Africa (Ohei et al., 2019; Schreck et al., 2020); many prospects have been lost as a result of such drawbacks. van Nieuwenhuizen (2012) identified that the quality of tertiary education had declined dramatically since 2008, which obstructed the quantity and the quality of advancement that the country could have experienced through work-ready graduates. Thus, South African graduate employability as an important HEI outcome has been emphasized (Ndebele and Ndlovu, 2019; Ohei et al., 2019). This study aimed to investigate the influence of the university environment and faculty on prospective university graduates and their readiness for employment.

## 2 Astin's Input-Environment-Output (I-E-O) theory

The university environments are the social variables, institutional interactions, interactions external to the institution, university administration, learning methods, and university infrastructure found within the higher institutions. Astin's Input-Environment-Output (I-E-O) theory 1993 stresses that students' academic development in the university is affected by academic inputs (relationship A), the educational environment (relationship B), and student output (relationship C). The factors of students' input, educational environment, and students' output as adapted and expanded from Astin's (1993) model are listed in Table 1 below.

**Table 1:** Academic Input, Environment and Output factors.

**Input Variables (relationship A):** Age, gender, ethnicity, residence, family income/socioeconomic, self-perception, status, parental educational level and parental expectation, individual variables, ability to learn, motivation, goals and commitment, prior educational experiences, prior schooling experiences, life circumstances, and academic involvement
**Institutional Variables (relationship B):** Mission and policy, budgeting and funding, institutional awareness and participation, academic variables, structural and normative system, social variables, mechanisms for social integration, institutional interactions, bureaucratic interactions, interactions external to the institution, university administration, learning methods, and university infrastructure
**Output Variables (relationship C):** Talents developed, graduation, career certainty, employment readiness, retention, dropout, skills acquisition, a record of academic achievements, and examination/test scores

I–O-E model investigates the implications of the relationship between academic input, educational environment, and academic output. Astin tested the postulation that learners' outcomes increased through a great study environment coupled with academic input. Based on the I-E-O model, Knight (1994) concluded that changes in policies in the institution (environment) positively influence the student's career choices and outcomes. Input can affect output either directly or by the interaction with environmental variables. Therefore, all these three variables have effects on each other. I-E-O model has been applied in studies to discern the relationship between learners' input, environmental factors, and students' outcome (see, for example, Astin and Sax, 1998; House, 1999; Pusztai, 2014; Espinoza-Parra and Collins, 2018; Hoh et al., 2018; Mukhopadhyay and Tambyah, 2019). This theory has been used to analyze learners' general academic experience, but it has not been used in the context of graduates' perceived employability and work readiness.

With the adoption of Astin's model, scholars found that the perception of skills in personal and academic development before and after the university was a significant contributor to self-confidence acquired after university (Mukhopadhyay and Tambyah, 2019). Gó mez and Su á rez, 2020 demonstrated that a supportive school environment enhances students' success and the quality of teaching practices predicts learners' educational outcomes. Similarly, Knight (1994) postulated that academic environmental influences determine whether students obtained desired educational results.

The I-E-O model deals with assessment and evaluation issues in higher education, including student learning satisfaction, employer satisfaction, and other outcomes (Hoh, Khattak, and LI, 2018). House (1999), who also applied the I-E-O model in his study, established that environmental variables did have a causal effect on students' success and satisfaction. Although this model offers a classification scheme to evaluate how students' characteristics (relationship A) and the university's program attributes (relationship B) may influence the desired results of graduates' programs (relationship C) - it is important to note that for this study, only the output and the environmental variables from Astin's theory were used. This is because the study only assessed the correlation between prospective graduates' perceived readiness to work (output) and the university's role in equipping students to engage in the world of work (environment). Hence, students' input variables of Astin's theory were purposely excluded in this study.

The study's primary emphasis is on prospective graduates' output (skills development and employment readiness); thus, it excludes graduated students and employers. The rationale for this is that it is critical to ascertain future graduates' views on their acquired abilities throughout their time in higher education. The focus on prospective graduates is required to comprehend how this set of students views themselves as employment-ready citizens, which influences their subsequent career paths. Additionally, placing a premium on potential graduates will aid in identifying areas in which the university environment may need to concentrate further to create new methods and practices that better match the labor market's requirements while students are still in the learning environment.

This study adopted I-E-O due to its relation to the sociological perspective, which maintains that our environment impacts our behavior. This theory can be referred to as an impact theory in that the foundations of which learners’ change result from environmental sources that affect the individuals. Therefore, instead of evaluating educational performance in the context of institutional popularity and resources, high-quality universities can assess the extent to which their roles boost graduates' employability and personal work readiness.

## 3 Review of literature

### 3.1 Graduate work-readiness and employability

Globally, employability and work readiness are concepts that are increasing priorities in the labor market and higher education environment. Work readiness is often used synonymously with employability, which focuses on an individual's potential to acquire desired employment. Both concepts vary based on the context and engagement of decision-makers and jobseekers (Geera and Onen, 2019; Wakelin-Theron et al., 2019). There is no unifying definition of graduates' work readiness. Still, it is used to denote a mix of qualities that differ around the globe between the categories of Higher Education Institutions (HEIs) (Keogh, Maguire, and O'Donoghue, 2015), and employability in the scope of university education refers to the mechanism by which academic institutions train students to navigate graduate activities and employment (Bennett, 2019). The basic concept of graduate employment readiness is a collection of similar features exhibited by skilled and capable learners as they continue to learn even after joining the workforce.

Borg et al. (2019) revealed that graduates' work-readiness profiles vary according to university and employer reputation. According to Caballero and Walker (2010), employers are increasingly valuing work-ready graduates - work readiness is regarded as a good indicator of a graduate's long-term job performance and career progression prospects. Given the rapidly changing nature of the workplace (Chigbu and Nekhwevha, 2021b) and the inflexible labor market (Allais and Nathan, 2014), the extent to which graduates are “work ready” (Caballero and Walker, 2010) with required skills reflect the perceptions of stakeholders regarding the work-readiness of graduates (Prikshat et al., 2019). For instance, employers have reported that they cannot find people with the right skills in South Africa (Allais and Nathan, 2014). These employers need skilled and credentialed graduates who exhibit the necessary characteristics to transition into current and future employment openings (Nankervis et al., 2019).

Employment preparedness attributes generally identify a range of widely predicted, transferable abilities that companies expect from workers (Shivoro et al., 2018). Essential aspects of employment prospects are communication abilities, strategic and logical thinking, collaboration, problem-solving, self-management, market and consumer understanding, initiative-taking, leadership and administrative skills, organization thinking, and job ethic (Tamrat, 2019). For McArthur et al. (2017), the most sought-after graduate qualities covered motivation, time management, communication abilities, and digital media expertise. These skills are complemented by emotional intelligence, creativity, and social skills (World Economic Forum, 2018). In the context of South Africa, “trainability and a willingness to learn, communication skills, creativity, ability to work with groups and conflict management were the top-ranked attributes expected of work-ready graduates” (Schreck et al., 2020, p. 52).

### 3.2 South African tertiary education and graduates’ readiness for employment

Morrison et al. (2006) argue that South African higher institutions will fail to function well if counseling and development centers to support students are not provided. The educational quality and its transformation in South Africa are gradually advancing to the expected level. The required development can be fully achieved by the way the educational system is organized, given that its structure determines the outcome of learning (Ohei et al., 2019). South Africa's education policy is influenced by the universal need for HEIs to be reactive to the expectations and requirements of the labor market, government, and society as a means of guaranteeing economic and social prosperity (Kruss, 2004). South Africans have much to achieve if they wish to take the quantum leap that is required to place them among the developed countries of the world; however, this is reasonably achievable with the right effort from the government, but the university system must be involved in the process (Tamrat, 2019).

The shortage of graduates with employable skills in South Africa is an inhibitor to accomplishing the government's potential economic growth target. For the South African economy to thrive to its highest, a HEIs environment must be of excellent standard and quality (Robertson, 2011). Some scholars objected to the responsibility placed on universities to work-ready their graduates.

In the words of Geera and Onen (2019, p. 12), *“while it is important to equip university graduates with employable knowledge and skills, the institutions can only provide generic skills. It is not necessarily the university's work to impart in the students' specific work-related skills since they are prepared for a wide range of employment options. Thus, specific job skills should be acquired while the graduate has already obtained employment. This means that even the employers must be ready to train their workers so that they can acquire those specific job skills”*.

Bennett (2019) suggested that the formation of employability is more successful in a process-based strategy in which the accountability for the development of employability can be shared amongst organizations, businesses, and students. However, universities need to note the current state of graduate employability from both employers' and graduates' viewpoints if they are to react successfully to each industry's expertise and skills requirements (Shivoro et al., 2018)**.** Mapping stakeholders' expectations would allow universities to empower their graduates with the employability attributes required by various industries (Shivoro et al., 2018).

### 3.3 Faculty influence on graduates’ career

The consequence of student-faculty interaction and influence is critical. Faculty influence focuses on teaching and learning that helps students grow intellectually and create new knowledge. This growth is cultivated through scholarly and creative accomplishments and professional educational programs outreach, which cultivate leadership in students to become contributing members of the global community. A student-faculty relationship may have a substantial impact on a student's success. According to Astin (1993), active learning with a faculty member has a robust and favorable connection to education and academic success, work readiness, and degree attainment. It can be argued that wide-ranging contact with faculty members contributes to student intellectual and social growth. The importance of learning and faculty knowledge development cannot be overstated for both students and faculty. Therefore, university bodies must be committed to offering the processes and encouragement for learners and faculty to be involved in meaningful interactions.

Brown and Krager (1985) specified that faculty members could consider the benefits and drawbacks of diverse personal development and job decision options, thereby promoting and securing the student's career decisions and, at the same time, recognizing individual learners as potential scholars. According to Super (1983), for individuals to make educational and professional choices, they need a helping hand to find the required competencies through experts such as university faculty members. It involves the faculty's commitment to interact with the students, encouraging them to be active learners in the classroom, and encouraging learners to seek informal contact with their teachers outside the class, thereby ensuring students' post-career choices.

### 3.4 South Africa's tertiary education and the economy

Globalization is forcing HEIs to meet the demands of the labor market, which increases competition amongst universities—meeting the market demands by creating learners who represent the knowledge economy (Froneman, 2003). There is a fundamental shift in the world's economic structure as the globalization of the labor market takes place and competitive forces are released across national boundaries. Globalization does not solely involve trade and economic expansion; it also means the forces and mobility of labor-power, learners' changing needs, and organizations worldwide (Froneman, 2003).

What the economy needs, what the organizations need, and what the graduates need in South Africa must be unified to engineer impeccable inclusive development. According to Raffe (2003), unification means that opportunities must be uniformly developed to advance lifelong learning, combining university curricula, vocational curricula, and post-compulsory learning and training systems into a unified system. In Robertson's (2011) words, "if South Africa is to provide wealth for its entire people, the country needs to attract and retain those who know how to create wealth and those who know how to assist them in creating wealth." Jenkins (1989, p. 2) attests that “because of the invidious combination of rapid population growth and economic stagnation, the gap between Sub-Saharan Africa and the rest of the world appears to be widening unless steps are taken to address the serious problems in education, this gap will in time become a gulf.” The argument by Robertson (2011) indicates that there is a correlation between knowledge, experience, economic development, and expansion.

Skills drive the economy and transform citizens' lives to become productive members of the workforce and the economy (Shivoro et al., 2018; Ndebele and Ndlovu, 2019). Effective modern economies require citizens with the aspiration and skills to study, set and achieve high standards, take responsibility for personal performance, and work cooperatively, with a strong foundation of general education, to develop and adapt to new knowledge and skills. HEIs' obligation to aid the growing nature of human capital development in the labor market has become a precarious issue in South Africa (Ngetich and Moll, 2013).

## 4 Methodology: study designs and participants

This study utilized a quantitative survey design strategy to decipher the influence of the university environment's role and faculty on prospective university graduates and their employment readiness. According to the university system, a university is composed of numerous faculties, and each faculty is composed of one or more professional schools. Faculty members manage traditional programs, emphasizing teaching, research, and academic growth. The university and its faculties are inextricably linked. This study concentrated on a university located in the Eastern Cape province of South Africa. The university is ranked 15th in South Africa by uniRank.org and ranked 3553 globally. The university has three campuses; however, the main campus with the largest population was selected to sample final year undergraduate students, including honours students, masters students, and doctoral candidates. The total population of eligible students at the campus was 5070. However, the number of students was separated in terms of their cohorts before sampling, with the total population in each segment as follows: final-year undergraduate students (2643), honours students (539), Masters students (697), and Doctoral candidates (326). The corresponding resultant sample size for each was 93, 82, 85, and 75, respectively, after the use of the Raosoft sample size calculator (@ http://www.raosoft.com/samplesize.html) with a 10% error margin, 95% confidence interval, and 50% response distribution, making the total sample size 335.

The study covered all genders with an age range of 19 years and above. Forty-point nine percent of respondents were in the faculty of Science and Agriculture, 29.0% in the faculty of Management and Commerce, while the respondents in the faculty of Social Science and Humanities were 20.0%. 7.8% were in the faculty of Education, and 2.4% in the Law faculty. 60.4% were females, and 39.7% were males, including South African and non-South African students. This subset of university learners was chosen because they contained smaller, manageable versions of the broader South African universities and campuses. It reflects other universities' learners' characteristics based on faculties, age, gender, and study level.

Concerning representativeness and generalizability, the sample size of 335 students from all faculties at this university represents the student population at this institution because the students share similar characteristics and operate at comparable social, academic, and emotional phases when placed in the same learning environment. As a result, the findings of this study are generalizable and representative of this learning group.

Two factors influence the study's representativeness and generalizability to other institutions in the nation or worldwide. First, various university types are defined by distinct patterns of student involvement in the institution's social and academic life. The variation in student involvement in particular campus activities that help determine their results is influenced by each institution's qualities and general environment (Chigbu and Nekhwevha, 2021a). Additionally, many institutional factors are associated with campus conduct differently among institutional kinds (Chapman and Pascarella, 1983). These distinctions may be ascribed in part to a university's instructional emphasis, content, and methods, as well as its surroundings (McManus et al., 2008). Due to the unique characteristics of each learning environment, our findings do not reflect or generalize across a cross-section of South African and international institutions.

Second, global and South African educational systems and environments have a common purpose. Higher education aims to create the quality and diversity of graduates needed for today's economy and the economy to which a nation aspires. The tertiary education sector offers social mobility and economic development pathways, leading to the global knowledge economy, from skills to immediate employment to building phases of complex learning to postgraduate study (World Bank, 2021). Regarding this function, where graduate learners' goals are to acquire skills, social mobility, and economic growth via a learning environment, this sample reflects other institutions in South Africa and extends internationally to some extent.

The list of students was obtained from the student administration office without direct individual identifiable information. Students' lists were released regarding data privacy and security, considering the data will be used only for research purposes. Before the direct data collection, consent was obtained from participants after explaining how the research would be conducted and the findings used. The student population was already divided into clusters based on different faculties. There are five faculties as identified above on the chosen campus of the university. As a result, a multi-stage cluster sampling strategy was adopted. In the first sampling selection stage, five departments in each of the five faculties were randomly selected. In the second stage, the researcher systematically chose samples from the pre-selected departments based on the proportion of completed students' size in each department. These steps were followed until the total sample of 335 participants was derived.

**Figure 1:**
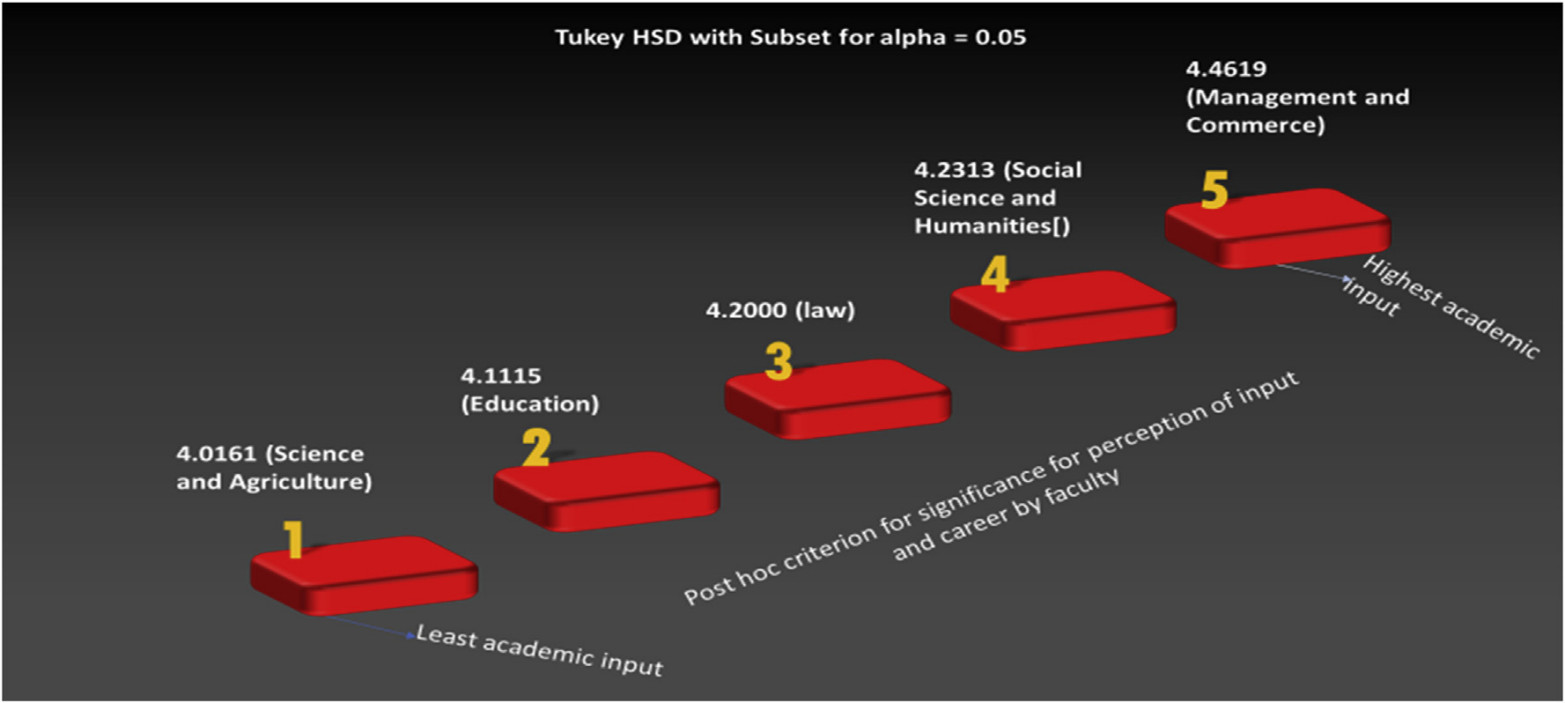
Morph animation: highest graduate input towards graduate skills by faculties. Source: Compiled by author.

The university education environment is meant to prepare promising graduates to be employment-ready. This will equip the students to confront the experiences they have in the work environment. Astin's theory was chosen because of its explanatory power to encompass the three variants of academic input, educational environment, and student output. However, Environment-Outcome (E-O) variants are central to the present study through a perception analysis. Against this background, the study laid the foundation for the hypotheses tested. Based on this study's theoretical framework, as depicted in Table 1 above, E-O variables were included in our self-designed questionnaire containing 51 items. Perceived Output, Skills, and Readiness (POSR), Faculty Influence (FI), and University Environment and Role (UER) were measured. Respondents were asked to rate their perception of the readiness for employment and how the university and the faculty as their learning environment have influenced their level of work readiness on measures of agreement and measures of capability ranging from “strongly disagree” (1) to “strongly agree” (5), and “Not at all Capable” (1) to “Very Capable” (5) (see Figure 1).

**Figure 2:**
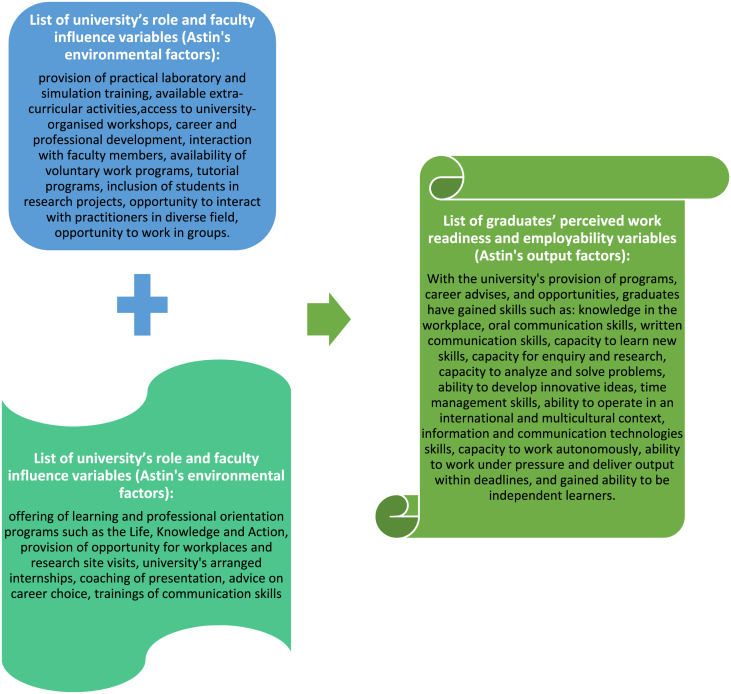
University and faculty variables and prospective graduates' perceived work readiness and employability variables. Source: Compiled by author.

The questions in this study considered how the completing university graduates appraised their perceived employability skills and how the university environment advanced their labor market preparation. As shown in Figure 2, the concepts included in the questions to ascertain the perceived acquired graduate labor market employability skills are varied. Initially, the study was piloted with 100 students to verify its reliability. A total sample of 30% of 335 students (n = 100) was used. The data was analyzed to determine if the proposed statistical tests were appropriate for the data collected. The pilot test directly answered all the research questions, including the study hypothesis, confirming the research instrument's validity. The University's Research Ethics Committee approved this study with an ethical reference number: NEK011SCH101.

## 5 Results

### 5.1 Hypothesis 1

**Ho:** There is no relationship between perceived output, skills, and readiness of prospective graduates and the university's role in equipping students to engage in the work environment.

**H1:** There is a relationship between perceived output, skills, and readiness of prospective graduates and the university's role in equipping students to engage in the work environment.

### 5.2 Hypothesis 2

**Ho:** There is no relationship between faculty influence and perceived output, skills, the readiness of prospective graduates.

**H1:** There is a relationship between faculty influence and perceived output, skills, the readiness of prospective graduates.

Correlation and multiple regression analysis were used to test if faculty influence and university environment and roles significantly predicted perception of graduates' output, skills, and readiness.

To test hypothesis 1, the bivariate correlations interpreted the relationship between the perceived output, skills, and readiness of completing students and the university's role in equipping students to engage in the work environment. In Table 2, POSR and UER were weak and negatively correlated, r (335) = -.119∗, p < .05. Despite that, the significance level is low; this result indicates that tertiary institutions equip learners with the competencies that different organizations are looking for. For this reason, the null hypothesis, Ho: is rejected, and the alternative hypothesis, H1, is accepted. Therefore, there is a relationship between the perceived output, skills, and readiness of prospective graduates and the university's role in equipping the student to engage in the work environment. Testing hypothesis 2, H1 in Table 2 indicates weak but positively correlated to POSR, r (335) = .123∗, p < .05 (see Table 3).

**Table 2:** Correlations of POSR, FI, and UER.

	POSR	FI	UER
POSR Pearson Correlation Sig. (2-tailed) N	1 335	.123∗ .024 335	-.119∗ .029 335

∗∗Correlation is significant at the 0.05 level (2-tailed).

∗Correlation is significant at the 0.01 level (2-tailed).

**Table 3:** Model summary.

Model	R	R Square	Adjusted R Square	Std. Error of the Estimate
1	.124	.015	.009	.55561

a. Predictors: (Constant), University roles, Faculty influence.

For this reason, the alternative hypothesis, H1, is accepted. There is a relationship between faculty influence and perceived output, skills, and the readiness of prospective graduates. Thus, this result concludes that faculty members influence students' skills and level of work readiness. To determine whether or not our model is a significant predictor of the outcome variable, We tested it using Model summary, Analysis of Variance, and Regression coefficients.

In this case, R = .124 shows a weak relationship, and the model indicates that the predictor variable can only explain approximately 2% of the variance in the data (R-square = 0.015).

The results in Table 4 show that the model was a significant predictor of perception of output, skills, and readiness, F (2,33) = 2.59, p = .036. As the significance value is less than p = 0.05, We can say that the regression model significantly predicts students’ perceptions of output, skills, and readiness.

**Table 4:** Analysis of variance - ANOVA.

Model		Sum of Squares	Df	Mean Square	F	Sig
1	Regression Residual Total	1.602 102.182 103.785	2 331 333	.801 .309	2.595	.036

a. Predictors: (Constant), University roles, Faculty influence.

b. Dependent Variable: Perception of output, skills, and readiness.

In Table 5, faculty influence contributed significantly to the model (β = .085, p < .05), university roles also did contribute (β = .053, p = .046). The final predictive model was: perception of output, skills, and readiness = 4.082 + (.085∗ faculty influence) + (.053∗ university roles). Cronbach's alpha scores showing scale reliability were reported. Any Cronbach's alpha score that is shown to be more than .70 is adequate (Hair, Anderson, Tatham, and Black, 1998). The POSR scale with Cronbach's alpha of 0.97, FI 0.90, and UER 0.82 were all reliable and accepted. Therefore, by calculating Cronbach's alpha coefficient, the scale's reliability and the questionnaires for this study were established (see Table 6).

**Table 5:** Model Unstandardized Coefficients Standardized Coefficients.

		B	std. Error	Beta	T	Sig
1	(Constant) Faculty influence University roles	4.082 .085 .053	.30413 .038 .074	.122 .039	13 .432 2.230 .714	.000 .026 .046

a. Dependent Variable: Perception of output, skills, and readiness.

**Table 6:** Post hoc analyses of faculty views on the perception of employment readiness.

Faculty	N	Tukey HSD Subset for alpha = 0.05			Mean	Std. Deviation	df	F	Sig.
		1	2	3					
Education	26	3.4642			3.4642	.35493	4	42.382	.000
Social Science and Humanities	67		3.8199		3.8199	.42549	330		
Law	8		4.0150		4.0150	.39408	334		
Science and Agriculture	137		4.1109		4.1109	.53388			
Management and Commerce	97			4.5514	4.5514	.08018			
Sig.		1.000	.155	1.000					

### 5.3 Comparing five faculty perceptions: output, skill, and readiness

Faculty views on the perception of output, skill, and readiness were compared among five faculties using one-way Anova. The 26 participants in the faculty of education had an average score of 3.46 (SD = 0.35); the 67 participants in the faculty of Social Science and Humanities had an average score of 3.82 (SD = 0.43); the 97 participants in the faculty of Management and Commerce had an average score of 4.55 (SD = 0.39); the 137 participants in the faculty of Science and Agriculture had an average score of 4.11 (SD = 0.53), and the 8 participants in the Law faculty had a mean of 4.02 (SD = 0.08). The scores in the different faculties were significantly different, F (4,330) = 42.38, p < .001. Post hoc analyses using the Tukey HSD post hoc criterion for significance indicated that the average scores for participants from Social Science and Humanities, Science and Agriculture, and Law faculties were not significantly different. In contrast, the participants' views differed from those of the other two faculties: Education, and Management and Commerce. Post hoc analyses on the university role on graduate students in each faculty show no difference in its influence. In summary, while graduate students from the faculty of Management and Commerce revealed that their faculty activities have significantly influenced their work readiness, prospective graduates from the faculty of Education received the least amount of graduate skills support from their faculty.

### 5.4 Measuring level of academic dedication by five faculties

Regarding the level of academic input that graduates from different faculties invest in acquiring labor market skills, the animation above showed that prospective graduates from the Faculty of Management and Commerce dedicate the highest academic input to achieve the maximum scholarly output needed in their studies and the workplace. Social Science and Humanities’ prospective graduates also revealed high levels of learning input in their studies. However, future graduates from the faculty of Science and Agriculture demonstrated the lowest level of educational input. The indication here is that the higher academic input prospective graduates receive, the more likely they will display confidence that they will be very competent in their intended career choices and with no doubt about efficiently working in any work setting. This is because they have devoted the highest academic input to their studies, coupled with adequate training and influence from their faculty, which has possibly prepared them for work where they can take responsibility for their own decisions and actions.

### 5.5 Relationships: Astin's academic environmental factors and academic output variables

The list of the university's role and faculty influence variables and prospective graduates' perceived work readiness and employability variables measured in this study are depicted in Figure 2 below.

The above figure depicts different academic activities and roles made available by the university and its faculties that the students believed to have impacted their work readiness and labor market abilities. For instance:

#### 5.5.1 Extracurricular activities

In the context of environmental variables and the students' responses to their work readiness, extracurricular activities were indicated as job-readiness influencers. Extracurricular activities refer to programs outside of the normal course of study at an institution. Still, the activities are organized around a specific aim, such as community service, educational clubs, hobbies, sports, and volunteer work. For example, extracurricular activities allow students to become active and connect with one another, resulting in improved learning and growth. Participation in such activities promotes social development by offering a forum for students’ engagement, connection creation, conversation, and awareness of others' differences and similarities, all of which are necessary for workplace readiness. Thus, extracurricular activities have proven to be great ways to supplement academic education, boosting students' educational outcomes.

#### 5.5.2 Group work experiences

Group work experiences were revealed to be another graduate work readiness influencer. Group work, or collaborative work, is when students work together in pairs or groups to build many of the abilities they will need in the future. Grouping students together on a project can help reinforce job-related abilities and strengthen communication and collaboration skills. Additionally, they can build competencies in delegating tasks and duties, pooling knowledge, and sharing various viewpoints. Teamwork is a transferrable skill that individual learners must possess to be job-ready and contribute towards achieving common goals in their future employment.

#### 5.5.3 Provision of student's internships

The results revealed that future graduates will benefit from the faculty-arranged internships. What is an internship? An internship is a term of work experience provided by a company to expose potential graduates to the working world, typically within a particular industry related to their subject of study. Internships provide students with the chance to explore and improve their careers, acquire new skills, build professional connections within the organization, and meet various professionals outside of the interned firm. Internships are a kind of early career development since they allow students to learn corporate etiquette, workplace cooperation, effective communication strategies, and personal traits. Students who have the chance to interact with industry professionals may obtain valuable business and industry insights, increase their knowledge of corporate culture, and also improve essential professional abilities such as good leadership. As a result, HEIs must provide more opportunities for students to participate in experiential learning and develop their competencies.

#### 5.5.4 Academic workshops

University-organized workshops were also indicated as one of the activities that impacted the work readiness of future graduates. Students participate in interactive academic workshops that analyze theories, promote a system thinking approach to learning, including practical case studies, and are led by a variety of specialists from academic faculties and industries. Also, universities provide a variety of programs and resources to assist postgraduate students in honing their research and thesis writing abilities. Through these workshops, students gradually gain confidence, participate freely in discussions, and become productive, which is beneficial to the student's intellectual development (Cilla and van Rensburg, 2015).

### 5.6 Descriptive statistics: patterns of graduate students’ responses

We computed descriptive statistics during the data analyses, which analyzed data that helped summarize the mean, median, and standard deviation (SD) constructively to show patterns of graduate students' responses that emerged from their views of the university's roles and faculties' influence on their employability. "Seminars and class presentations enhance oral communication skills needed in the workplace," mean = 4.56, SD = 0.702, is the most significant factor in describing respondents' perceptions of the university environment and their roles in skills. The main element in describing future graduates' perception of faculty influence on their career and skills is “Teaching, and learning members have an influence on my career choice,” mean = 3.49, SD = . .868. Class presentations and seminars are essential parts of academic life that benefit students in many ways. Students are given the opportunity to improve their communication and presentation abilities during these activities.

In summary, results show that higher education institutional factors in Figure 2 above, representing Astin's environmental factors, influence prospective graduates' perceived work readiness and employability variables identified in Astin's output factors of students' development.

## 6 Discussion

Active teaching and learning have risen to prominence as a priority throughout this period of pedagogical change. What is active teaching and learning? The concept refers to a diverse range of methods, including collaborative learning, extracurricular activities, seminars, workshops, internships, and group work presentations, that provide opportunities to develop additional skills in graduates. The results reveal that higher institutions influence graduate students' work readiness via collaborative learning, promoting graduate-faculty interaction and preparation for employment situations.

### 6.1 Seminars and departmental support: boosters of job-related skills

As Chigbu and Nekhwevha (2021a) assert, students often attach social life at university to their academic input and success. According to the students in this study, seminars were an effective mode of education because students were actively involved in critical thinking by posing questions about literary texts, discussing ideas, and honing their communication and presenting skills (Al’Adawi, 2017). Seminars have been linked to boosting job-related skills unique to each student in different fields. Support from departmental experts has also been linked to future graduates' expectations of their academic skills necessary for a successful transition into the work environment (Bhandari et al., 2013). Again, it was revealed that graduate students are actively involved in university arranged internships to gain the whole experience of their lifetime in terms of learning. These internships act as an entry ticket for students to be introduced to the already active world of professionalism. Also, according to Williams (2011), students learn more during group tasks.

### 6.2 High-quality learning environment: a greater level of learning

Based on this study, prospective graduates' labor market readiness depends on the university and their faculties in readying the graduates for professional life. Indeed, Chigbu and Nekhwevha, 2021a, Chigbu and Nekhwevha, 2021b elaborated that a positive and productive study environment significantly impacts a learner's academic progress. If an educational institution does not provide a diverse and high-quality learning environment for its students, it fails to fulfill its objective. Each study setting that students select is anticipated to affect their capacity to concentrate, focus, and persevere in the face of learning problems. For example, the ongoing teacher learning process, which involves understanding new pedagogies and teaching techniques, will assist students in attaining a greater level of learning (Chigbu and Nekhwevha, 2021a) and “robust and coherent skills development” (Allais, 2012, p. 1, p. 1).

Tertiary institutions' primary objective is to develop their students' intellectually to be morally, materially, socially competent citizens. Suppose the university leavers are well prepared to be academically skilled during their learning days, committed to achieving their career goals, motivated to go beyond reading and be involved in their studies in their prior educational experiences and representing their effort. In that case, they will not have difficulties confronting challenges in the new environments. Consequently, they will perform better, which is the outcome of their university education environment as the institution becomes responsive and flexible in the changing world (Lotz-Sisitka et al., 2015). The skills development assessment of quality stresses the effect of learning bodies on its learners. The indisputable evidence is that genuine quality is defined by the institution's ability to positively influence its learners, improve their intellectual growth, and create an encouraging change in their lives. This would entail an attempt to correlate the relationship between a particular activity and the level of social, cognitive, or academic development of their prior skills with the students' present perceived work readiness. University administrators should recognize that nearly all institutional rules and traditions could significantly affect how learners develop or spend their energy on being work-ready.

### 6.3 The level of graduate's work readiness varies by faculties

Our study also found that graduate students' levels of work readiness vary due to the level of faculty and university support they receive. How higher education institutions arrange themselves determines their learners' quality of learning, readiness, skills, confidence, and career attitude. Completing graduates assume that the HEIs would have the requisite preparation to equip them with invaluable employability skills and knowledge compulsory for the workplace (Mari et al., 2019). While some prospective graduates highly rated their faculty support in influencing their perceived graduate level of employability, a good number of graduates were not academically impacted by their teaching and learning members. These unsatisfactory ratings are why many scholars have unanimously maintained that universities and their faculties have lagged in accomplishing employability responsibilities. Shivoro, Shalyefu, and Kadhila (2018), Bennett (2019), Prikshat et al. (2019), and Wakelin-Theron et al. (2019) conclude that universities have increasingly struggled to build on this responsibility, allowing other knowledge providers to address students' and businesses' demands. Brown and Scase's (2005) stressed that completing learners' quality from a particular faculty affects transitions into employment.

### 6.4 Practical university's roles and scholastic achievement

As the findings indicate, university learners' perceived employability aligns with the university's role in equipping students to engage in the work environment. Scholars across the globe (Harvey, 2000; Archer and Chetty, 2013; Bennett, 2019; Mari et al., 2019; Ndebele and Ndlovu, 2019; Ohei et al., 2019; Okolie et al., 2020) are all in agreement that HEIs have a decisive function to perform in the development of academic graduates with practical abilities, qualities, consistent expertise, and practice in the work environment. In alignment with the result of this study, Mari et al. (2019) maintained that graduate employability is mostly the university's responsibility compared to employers and students. Future graduates anticipate everything they have studied at school to be applied to their actual careers (Mari et al., 2019). As it has been stated, “an excellent and effective study environment is what makes the most remarkable difference in the learner's scholastic achievement” (Chigbu and Nekhwevha, 2021a, Chigbu and Nekhwevha, 2021b, p. 1).

### 6.5 Study limitations

We acknowledge two limitations in this study. 1) this project excluded the perception of graduated students and employers, which limited the results of this study. The methodology section above explains the rationale for excluding graduated students and employers. 2) This study only employed a quantitative data gathering approach. Combining quantitative and qualitative studies would have shed more insights that could not be learned from only quantitative studies. Nonetheless, the results of this study are reliable, valid, and rich in crucial implications.

## 7 Conclusions and recommendations

Levels of skill inequality substantially differ across faculties. The disparity in skills development, outcomes, and opportunities can be explained by the faculty's non-inclusive collegiate skills preparation for the world of work. Given the industrial 4.0 facing many organizations amid job automation, significant impacts on the graduate skill revolution are expected. While graduate skills provisions offered by university faculties differ, they invariably affect graduates' employment readiness and skills outputs. The pedagogical activities related to extracurricular activities, internships provided by universities, group work experiences, university-organized workshops, and seminars impact the enhancement of employability and efficiency of skills development of graduates. Faculty influence is at the heart of graduate students' learning opportunities; thus, the quality of the influence is of paramount importance.

Contextualized accounts consider learners and their environment as components of a unified whole. As such, it is critical to approach universities from a systems viewpoint to comprehend the entire (university environment), the parts (faculty and students), and their interactions (skill development, labor market readiness, career progression, and opportunities). Learning development is an interacting system of institutional settings and the students' output. The individual's academic output consists of skills acquisition, capabilities, a record of academic achievements, talents developed, graduation, employment readiness, and expertise of the graduate student. These outputs are directly influenced by the academic environment, which encompasses institutional awareness and participation, social integration mechanisms, educational interactions, infrastructure, mission, policy, interactions external to the institution, university administration, learning methods, and faculty interaction (Astin, 1993; Chigbu and Nekhwevha, 2021a).

The inability of a university environment and its faculties to skill their graduate students will lead to unemployment, underemployment, missed recruitment, and fewer economic development opportunities to build human capital while studying. However, the immediate consequences can result in other short- and medium-term effects that may be just as severe in the long run. For instance, increased poverty and inequality. Graduates with a high level of competence are better equipped to deal with the disequilibria created by events such as altering labor processes and job transitions as a result of the technology revolution and adoption (Chigbu and Nekhwevha, 2020) because they can adapt to the changing needs of new technologies, employers, and the future of work.

Thus, it can be said that higher education is a stock. This stock is owned by graduates, employers, HEIs, and the government. If there is any considerable profit from this stock, all parties benefit. As argued by Borg et al. (2019), the institutions that educate students who go on to work in the Industry 4.0 workplace, the students themselves, and the industry that encompasses all organizations and employers with whom the graduates seek employment are three key stakeholders who bear responsibility for students' readiness to work in Industry 4.0. All of these stakeholders profit from graduate work readiness collaboration.

In the short term, it is therefore crucial for tertiary institutions to support the development of skills that foster individual graduates' resilience by meeting the demand from labor markets. In the medium run, universities and their faculties will need to adapt to a rapidly changing pedagogical landscape. Interventions will need to be adjusted in line with the future of work and be tailored to each university's faculties and departmental structure. Finally, to ensure a longer-term intervention, faculties must address pre-existing pedagogical challenges that are likely to accelerate and become more urgent in light of the changing nature of the labor market. Among these is the provision of adequate support to all prospective graduates through practical graduate-level skills training, retraining, and collaborative, constructive evaluation of skill level ratings and feedback.

Now is the time for universities, faculties, and members to reposition themselves as the most influential driving forces for graduate skill development and create new and more inclusive pipelines for the most susceptible prospective graduates to contribute to the global skills demand. This study represents a bold call to more action for HEIs to prepare, support, and develop completing graduates for the present and future of work.

## Declarations

### Author contribution statement

Bianca Ifeoma Chigbu: Conceived and designed the experiments; Performed the experiments; Analyzed and interpreted the data; Contributed reagents, materials, analysis tools or data; Wrote the paper.

Fhulu. H. Nekhwevha: Conceived and designed the experiments; Contributed reagents, materials, analysis tools or data; Wrote the paper.

### Funding statement

This work was supported by National Research Foundation (NRF) under Grant (number 90414).

### Data availability statement

The authors do not have permission to share data.

### Declarations of interests statement

The authors declare no conflict of interest.

### Additional information

No additional information is available for this paper.
